# Going to scale: design and implementation challenges of a program to increase access to skilled birth attendants in Nigeria

**DOI:** 10.1186/s12913-017-2284-2

**Published:** 2017-05-18

**Authors:** Edward N. Okeke, Emma Pitchforth, Josephine Exley, Peter Glick, Isa Sadeeq Abubakar, Amalavoyal V. Chari, Usman Bashir, Kun Gu, Obinna Onwujekwe

**Affiliations:** 1 RAND Corporation, 4570 Fifth Avenue, Pittsburgh, PA 15213 USA; 2 RAND Europe, Cambridge, UK; 3 Bayero University Kano, Kano, Nigeria; 4 University of Sussex, Brighton, UK; 5 University of Nigeria Enugu, Nsukka, Nigeria

**Keywords:** Skilled birth attendance, Implementation, Midwives, Nigeria

## Abstract

**Background:**

The lack of availability of skilled providers in low- and middle- income countries is considered to be an important barrier to achieving reductions in maternal and child mortality. However, there is limited research on programs increasing the availability of skilled birth attendants in developing countries. We study the implementation of the Nigeria Midwives Service Scheme, a government program that recruited and deployed nearly 2,500 midwives to rural primary health care facilities across Nigeria in 2010. An outcome evaluation carried out by this team found only a modest impact on the use of antenatal care and no measurable impact on skilled birth attendance. This paper draws on perspectives of policymakers, program midwives, and community residents to understand why the program failed to have the desired impact.

**Methods:**

We conducted semi-structured interviews with federal, state and local government policy makers and with MSS midwives. We also conducted focus groups with community stakeholders including community leaders and male and female residents.

**Results:**

Our data reveal a range of design, implementation and operational challenges ranging from insufficient buy-in by key stakeholders at state and local levels, to irregular and in some cases total non-provision of agreed midwife benefits that likely contributed to the program’s lack of impact. These challenges not only created a deep sense of dissatisfaction with the program but also had practical impacts on service delivery likely affecting households’ uptake of services.

**Conclusion:**

This paper highlights the challenge of effectively scaling up maternal and child health interventions. Our findings emphasize the critical importance of program design, particularly when programs are implemented at scale; the need to identify and involve key stakeholders during planning and implementation; the importance of clearly defining lines of authority and responsibility that align with existing structures; and the necessity for multi-faceted interventions that address multiple barriers at the same time.

**Electronic supplementary material:**

The online version of this article (doi:10.1186/s12913-017-2284-2) contains supplementary material, which is available to authorized users.

## Background

The lack of availability of skilled providers in low- and middle- income countries is considered to be an important barrier to achieving reductions in maternal and child mortality in developing countries [13, 14]. Studies estimate that up to a third of maternal deaths and half of neonatal deaths could be prevented by increasing rates of skilled birth attendance [11]. However, there is limited research on programs increasing the availability of skilled attendants in developing countries, in particular, programs that have been implemented at scale. We study one such program in Nigeria.

This program, known as the Midwives Service Scheme (MSS), was introduced in 2009. The main objective of this program was to increase coverage of skilled birth attendance in rural communities in order to reduce pregnancy and birth-related maternal and child mortality. Nigeria is the second largest contributor to maternal deaths globally and accounts for approximately 14% of all deaths [23]. In 2008 the maternal mortality ratio was 545 per 100,000 live births nationwide, and more than 800 per 100,000 births in rural areas. Only 39% of births in 2008 (28% in rural areas) were attended by a skilled attendant [16]. One in three women cited provider availability as a serious problem in accessing health services [16].

The MSS was designed to address these barriers. The main feature of the program was the recruitment and deployment of midwives to primary health care facilities (four midwives per facility to provide 24-h coverage). It, however, also included supply of basic equipment and supplies (such as blood pressure apparatus, stethoscopes, and essential drugs and consumables), and strengthening of community involvement via the formation and reactivation of Ward Development Committees. These are made up of influential people in the community and meet monthly to discuss health and other developmental issues in the community [1]. The MSS was based on a hub and spoke model in which four primary healthcare facilities with the facility to provide basic essential obstetric care were clustered around a general hospital with the capacity to provide comprehensive emergency obstetric care.

The MSS was funded by debt relief funds under a 2009 Appropriations Act and was designed to be a collaborative effort between the Federal, State, and Local Governments. Under the memorandum of understanding for the program, the federal government was supposed to recruit and deploy the midwives, pay them a monthly allowance of N30,000 (approximately 200 USD at the time), and supply clinics with basic equipment and drugs. State governments were supposed to make upgrades to referral hospitals, provide monitoring and supervision, and pay midwives additional allowances of N20,000 monthly. Finally, local governments were supposed to provide free housing for the midwives and contribute an additional N10,000 a month to their salaries. The memorandum of understanding also included a plan for program midwives to be absorbed by state governments into regular employment [18].

In the first phase of the program 2,488 midwives (slightly short of the expected 2,608) were deployed to 652 primary health care clinics (PHC) across Nigeria’s 36 states and one Federal Capital Territory. Nigeria’s six geopolitical regions were classified as moderate maternal mortality (south east and south west regions), high maternal mortality (north central and south south), or very high maternal mortality (north east and north west) and this determined the number of participating facilities. There were 12 PHC facilities per state in moderate mortality regions, 16 PHC facilities per state in high mortality regions, and 24 PHC facilities per state in very high mortality regions. The participating PHC facilities had to meet various criteria including being located in a hard-to-reach or underserved community, offering 24-h health services, and possessing a minimum set of equipment and basic laboratory facilities. Participating midwives signed one-year contracts, renewable subject to satisfactory performance.

The MSS is of policy importance because it is a rare example of a program designed to increase the availability of skilled birth attendants that was implemented at scale. It has been highlighted as a model for other sub-Saharan African countries [9]. Despite its promise, however, an impact evaluation carried out by this team found that the MSS had only a modest impact on the use of antenatal care and no measurable impact on skilled birth attendance [20]. This paper draws on in-depth interviews conducted with federal, state, and local government policymakers responsible for implementing and running the program, and with program midwives, and also draws on focus groups conducted with community residents to try to understand why the program failed to have the desired impact. It provides important lessons for policymakers in other countries interested in scaling up skilled birth attendance programs.

## Methods

We conducted semi-structured interviews with federal, state and local government policy makers and with MSS midwives. We also conducted focus groups with community residents including members of ward development committees, women of reproductive age, and men.

We chose three states for the study: one from a moderate mortality region (Enugu state in the south east), one from a high maternal mortality region (Kwara state in the north central), and one from a very high maternal mortality region (Kano state in the north west). We chose states with different maternal health utilization and population characteristics (see Table 1) and based on the presence of partners in these states. In each state we chose three primary health facilities as fieldwork sites. The facilities were purposively selected to capture a diverse range of health facility characteristics including facility infrastructure, number and mix of staff, number of deliveries, and facility-level mortality (see Table 2). The final selection also took into account pragmatic reasons such as travel requirements, and safety/security of field personnel.

**Table 1:** Selected characteristics of chosen states before introduction of MSS

	Antenatal care from skilled provider	Birth attended by skilled provider	Female literacy rate	Predominant Religion
Enugu	68%	66%	73%	Christian
Kano	50%	13%	31%	Muslim
Kwara	58%	53%	48%	Muslim
National	58%	39%	54%	-

Note: Data is from the 2008 Nigerian Demographic and Health Survey

**Table 2:** Overview of clinic characteristics

State	PHC	Resources	Days open per week	Staff			Number of reported deliveries (July to Dec. 2013)	Number of infant deaths (July to Dec. 2013)
				Number of doctors	Number of nurses	Number of midwives		
Enugu (E)	1^a^	No reliable electricity	7	1	0	4	28	0
	2	Has electricity	7	0	2	4	20	0
	3	Has electricity	7	1	0	1	27	1
Kano (K)	1	No reliable electricity	5	0	0	4	925	358
	2	No reliable electricity	7	1	0	2	176	n/r
	3	Has electricity	5	1	0	3	240	0
Kwara (Kw)	1	Has electricity	7	0	1	3	2	0
	2	Has electricity	7	0	0	5	40	0
	3	Has electricity	7	0	0	5	59	0

Note: n/r = no records. ^a^ This facility was from a new updated version of the program that included additional program components such as facility upgrades

### Semi-structured interviews

We identified policy makers based on their involvement in the scheme, using official websites and the study authors’ professional networks. For the midwife interviews, we randomly selected two midwives in each health facility. Potential participants were approached by the researchers, who explained the purpose of the study and answered any questions before seeking verbal consent. Participants were informed that participation was voluntary and they could withdraw at any time without giving a reason. The interviews were undertaken by locally trained researchers in English or the local language. Interviews with policy makers were conducted in a place of their choosing, while the interviews with midwives were conducted in the vicinity of the health facility, although effort was made to hold them in a space away from the facility to reduce interruptions and allow the participant to speak freely. The main focus of the interviews was to understand perceived barriers and facilitators to the success and long term sustainability of the program. We pretested interview guides in the selected states to ensure cultural sensitivity. The interview guides used are included as Additional file 1.

### Focus groups

The focus group discussions were organized by type of participant (women, men, and community leaders), to help ensure that participants were able to talk freely. The main objective was to understand perceptions and experiences with the program. Potential participants were identified with the help of a village guide, who was also responsible for convening the groups. Focus groups were held at a time and place convenient for participants. Participants were compensated for their travel and refreshments were provided. Focus groups followed a semi-structured format.  The focus group discussion guides are included as Additional file 1. Two facilitators moderated the focus group discussions to ensure smooth running and also to record interactions within the group. With the consent of participants, interviews and focus groups were recorded and later transcribed verbatim and translated into English by the locally trained researchers. Formal back translation was not undertaken but the transcripts were reviewed by the coordinator in each state for accuracy.

Interviews and focus groups were conducted between November 2014 and January 2015.

#### Data analysis

The interviews and focus group data were analysed together in QSR Nvivo software based on the constant comparative approach [8]. The data were read and re-read and initial ‘open codes’ were applied to the data by two independent researchers. These were incrementally grouped into organizing categories, or ‘themes’, which were modified and checked constantly in order to develop a coding frame with explicit specifications. The coding frame was agreed by both researchers. The coding frame, influenced partly by the research questions but also by ideas arising during the data collection, was used to systematically assign the data to the thematic categories [3, 21]. Anonymized quotes from participants have been used to illustrate key themes below. Respondents and focus groups are identified first by the state (E = Enugu, K = Kano, Kw = Kwara) and clinic (1, 2 or 3), and then by the participant type (FPm = Federal Policymaker, SPm = State Policymaker, LPm = Local Government Policymaker, M = Midwife, FG = Focus group).

## Results

In total we conducted 33 interviews with 16 midwives (in two health facilities it was possible to recruit only one midwife) and 17 policymakers (three federal, five state and nine local). Summary characteristics for interviewed midwives are presented in Table 3. Because of the small sample size, to preserve confidentiality we do not report characteristics of policymakers. We conducted nine focus groups: three in each state (one each with community leaders, men, and women). The number of participants in each focus group ranged from five to nine. Summary characteristics for focus groups are presented in Table 4. Below we highlight the key program challenges as perceived by the various groups. We first present policymakers’ perspectives, and then we present midwives’ perspectives. Where relevant we augment with findings from the focus groups of community leaders, women and men.

**Table 3:** Summary characteristics of interviewed midwives

Midwife	Clinic	Level of experience prior to entry	Program Tenure
E1M1	1^a^	In retirement	1 year 8 months
E1M2	1^a^	Worked for a year in private clinic	9 months
E2M1	2	Year compulsory youth service	5 years
E2M2	2	In retirement	3 years 9 months
E3M1	3	Newly qualified	4 months
E3M2	3	Year compulsory youth service	1 year 4 months
K1M1	1	Over 18 years	4 years 7 months
K1M2	1	Newly qualified	4 years
K2M1	2	Newly qualified	10 months
K2M2	2	Newly qualified	4 years
K3M1	3	In retirement	4 years
Kw1M1	1	In retirement	5 years 3 months
Kw1M2	1	Year compulsory youth service	3 years
Kw2M1	2	In retirement	5 years
Kw3M1	3	In retirement	4 years
Kw3M2	3	In retirement	2 years

^a^This facility was from a new updated version of the program that included additional program components such as facility upgrades

**Table 4:** Focus groups

FGD	PHC	Participant	Number of participants
E1FG1	1	WDC	7
E2FG2	2	Men	7
E3FG3	3	Women	8
K1FG1	1	WDC	9
K3FG2	3	Men	5
K2FG3	2	Women	5
KW1FG1	1	WDC	8
KW3FG2	3	Men	8
KW2FG3	2	Women	8

Note: We only have age data for two groups, E2FG2 and E2FG3, where the average age of participants was 51.7 and 29.8 years respectively

### Policy makers’ perspectives

#### There were problems with the design of the program

Several state-level policymakers suggested that the design of the MSS was flawed from the beginning because it did not sufficiently take into account differences across states. These policymakers attributed this to lack of consultation during the design stage.

*‘You cannot stay in Abuja and then take into consideration the peculiarities of different states and this design is designed in a generic form, so they designed it in a generic form without a recourse to the peculiarities to different states, different LGAs [local government areas], different cultural background, different educational level, different socio economic level, they don’t have recourse, they just design it in generic form in Abuja and then force down the throat of states’* (ESPm1)

*‘The number of health facilities selected cannot cover. They are grossly inadequate. They didn’t take into consideration the size or population of the states. For example in Kano State, we have 44 LGAs, about 1300 health facilities, 27,233 settlements. The number of health facilities cannot cover. There is an issue right from conception. Kano with 44 LGAs is given the same number of health facilities with a state that has only 8 or 11 LGAs with maybe 400 or 200 facilities. This creates inequity right from the beginning. Instead of the planners to seat down with each state and determine each state’s individual requirement, they just blindly allocated as they wished. There may be some significant improvement in some other states. Even if there is, maybe in small pockets within some communities, but this is not reflecting in the overall state picture. So there may be significant achievement in some states but not in Kano, where only 36 out of 1300 operate MSS. With that we can only achieve about 10% success.’* (KSPm1)

Another problem highlighted by state-level policy makers was the fact that the program required midwives to be posted to states other than their states of usual residence. For the majority of state-level policymakers this requirement that midwives be relocated was perceived as one of the major threats to the long-term sustainability of the scheme.

*‘The way recruitment is structured, the thing was designed just like the volunteer teachers scheme that we developed in Enugu state, you work in the community where you retired from so that logistics problems will not arise, but you will see one of the midwives being posted from Enugu state to Borno, Enugu state to Adamawa, how do you think they can function there defeating the aim you know using the catchment area.’* (ESPm3)

*‘Sustainability will affect especially in the north. In the north we do not have enough indigenous midwives, we rely on southerners and this creates a problem. Retention rate is higher in the south because they are at home there. Many of them working in the north after sometime will tell you they want to go back home because they want to go back to their communities to marry or so.’* (KSPm1)

This issue was also mentioned by community residents in the focus groups.

*‘The nurses should be devoted to their work. Most of them are married and are not living with their husbands. They go to visit their husbands and as such are not usually around. But, they try the way they should. […] that while sending these midwives to the rural areas, they should be sent to their state or their local government where they will be close to their husbands, so that they will concentrate and do their work well.’* (E3FG3)

Another design issue brought up by policymakers was with the division of responsibility for the scheme. In their view it did not align with the organization of health care in Nigeria. For example, they noted that the MSS was a primary health care program but it involved the state governments whose responsibility is supposed to be delivery of secondary health care, whereas primary care is normally the responsibility of local government

*‘Not every state even if entire state adopts its state primary health care…establishes its own primary health care development agency, the issue of where does which one belong to is still a problem, we know that primary health care facilities is under the control of the local government, secondary health facilities are under the control of the state and tertiary health care facilities are under the control of federal government. So the same way, this issue of who is in charge, who is in control’* (ESPm2)

The contract nature of MSS midwife positions was also seen as an important challenge. One state policymaker in Kano, for example, noted that MSS midwives and state employed staff were not treated as equals by the state government, leading to lack of training opportunities or promotions for MSS midwives.

*‘If you look at these midwives, it is not possible for you to retain them for a long time on contract basis. Sustainability may be better if the states take over completely, since the midwives will now be absorbed as permanent staff in accordance with the MOU [memorandum of understanding]. It is there in the MOU that the midwives should be absorbed, but even in Kano State, when the scheme was started, there was no establishment of the Board, but now that there is establishment of the board, we will attempt to absorb them because the board is more technical than the Ministry. Employ rather than continue to use them as contract staff. If you leave them as contract staff for a long time, they are more likely to leave the service and go elsewhere. However, unfortunately many of the States do not have a policy for absorbing, even in Kano. However, offering them full time employment will be a challenge since when we attempted to do so in Kano, the Ministry of Health was saying they will send them to secondary facilities, because they don’t have midwives there too.’* (KSPm1)

*There was insufficient “buy-in” by state and local governments*

One issue raised by federal policymakers was that states and local governments did not consistently do their part – paying midwives their allowances and providing adequate housing. They noted, however that, this was not the case in all states.

*‘[…] so some midwives salaries would not be paid by the States for a long time. It [being paid] a huge issue in some places and not in others’* (FPm1)

*‘Some of the states are very good, extremely good, you know, like they take care of the midwives, they pay their own aspect, because we have what we call a memorandum of [understanding]… between us and the states… so where the state is expected to pay at least another 30,000 on top of what we pay the midwives. And then pay something for the local government, so to do that religiously, every month, so the midwives are compensated for being ready. But some states, well, you know, they are not even bothered, you know, to do it.’* (FPm2)

This irregular payment of salaries was perceived as having a negative impact on midwife morale and their willingness to continue with the program.

*‘Regular payment of the stipends will make the scheme sustainable, but when they stop paying them the stipends, the morale of those involved will dampen. […] Whatever you do is measured in terms of money, and the more money you are given, the more work is expected of the person, so the present allowance should be improved.’* (ELPm3).

State and local government policymaker interviews revealed a deep sense of dissatisfaction with the limits to the extent of their involvement during the planning and implementation of the program. There was resentment particularly in Enugu, towards the federal government, whom they considered to have forced the scheme upon states without adequate consultation.

‘*The concept is a good program but the implementation, the implementation I will tell you has jaundice […] if you want this scheme to be sustained, definitely you should involve the people that need it and therefore the design, and the implementation should be bottom-top approach, where even the communities are part of the design, the LGA is part of the design, the states are part of the design, but if you come to hang it at the national level, definitely the state will leave, it is not something that is designed at that level is sustaining, it is difficult to sustain anything that is hung at that level.’* (ESPm1)

*‘For it to go well, we have to return to the drawing table because it is a good scheme, it is a good scheme, we have to actually return to the drawing table and em explain to the stakeholders what you are doing, explain to them how you are funding it, explain to them how you disbursing the funds, carry them along when you are doing the recruitment, because at the local government level where you are going to post those people to the community, there are indigenes there who I know will be better positioned to run those program, you can imagine posting somebody from Enugu to Adamawa state, do you think the person will go, he will just sign acceptance letter and stay where she is, so there must be a proper planning, I don’t think they have planned enough in implementing the program.’* (ESPm3)

*‘They [Federal government] do everything, as a matter of fact most state don’t even know what is happening in MSS, they hire they pay, they supervise without involving the state, I find, one still wonders how somebody in Abuja will be able to supervise an employee in Enugu or say any other state in Nigeria, […] so as a matter of fact we have no hands because we cannot supervise people you did not hire.’* (ESPm2)

*The scheme did not address other important demand and service delivery constraints*

Many policymakers who we interviewed cited other important barriers that the program did not address. Lack of transportation, both for members of the community trying to reach a health center and from a service perspective for midwives to get to women in the community, or for referral between health facilities, was cited as an important constraint that was not addressed by the program.

*‘The second challenge is poverty, it will be the problem, because most of them, they don’t have means, number one, if you take this transportation, that is, the challenges that we have been facing. We have facilities, like here in [PHC name] but you have a lot of ahh…patients/clients all over surrounded to the area, but they cannot have any means of transport to be here. So it is one of the greatest challenges.’* (KLPm3)

*‘The staff are working with nothing, no good equipment, no services like Van or Maternal Child Health buses. The government of Enugu state bought Maternal Child Health buses and distributed to all the health departments, most of these buses have since been dilapidated and are no longer good to go on in the services. Somebody could be in labor and there will be no vehicle to transport that person to the next existing level of health care, so it is one of the big problems facing us.’* (ELPm2)

Another important constraint noted by local government policymakers was the lack of education/awareness about the importance of receiving proper pregnancy and childbirth care.

*‘So the greatest challenge of this maternal child health, I can say ahh, social mobilization in the first place, because many people, many clients, they don’t know what is happening and related to this antenatal services. So it is when we were, they were informed what is the importance of this antenatal care, they normally try to be in the facilities with the conduct of these services.’* (KLPm3)

### Midwives’ perspectives

For most MSS midwives, the primary motivation for participating in the program seems to have been one of vocation and having a rewarding job in which they could help others, referring to things like, *‘it is service to humanity’* (E2M2)*,* and ‘*to save life and to help the nation’* (K1M2)*.* Several midwives reported that their driving motivation was the reward from helping others rather than any financial rewards.

*‘Because, this field is a kind of vocational field. It is a call and a volunteer work. It is not based on the income or what you see. It is just to help and render humanitarian service to people, and to save life.’* (E3M2)

For a few midwives, however, participation in the scheme was simply a means to an end.

*‘Midwifery service scheme has me a temporal job. I don’t think there is any other thing.’* (E2M1)

*‘I’ve worked with the government, and after retiring I was not tired, and I wanted to assist our women during childbirth and the rest.[…] I am telling you this work I am doing, it is because I don’t want to just sit at home. Truly, there is not anything good about it.’* (K3M1)

Many of the midwives we interviewed perceived their work as having had a positive impact in the community.

*‘Working in the facility, MSS have contributed a lot in women’s health by rendering quality and focused antenatal care to them, conducting sound and healthy labor for them, administering sound family planning to them, educating them mainly during antenatal care on how to take care of the their babies and children, like the nutritional status, personal environmental hygiene and preparing for their birth issue, and also administering family immunization to their children and follow up home visits and all other things.’* (E3M2)

*‘Before we came, there was low attendance at ANC and delivery at the facility. But when we came, we do community mobilization and those that came to the facility are appreciating our work. We’ve reached almost 80% of attendance.’* (K1M1)

Despite these benefits, however, the midwives identified a number of important operational barriers and challenges.

#### Human resource constraints

A challenge noted by midwives was that there was inadequate staffing of health centers. They perceived this as resulting in having to work longer hours.

*‘Wow! To God Almighty, sincerely my feeling towards this job is 150% not even 100% guarantee because even when I am off duty I come out to volunteer and do the job. There was a day I was preparing for church and I was off that day, I saw a woman on labor and nobody was in the hospital to assist her, I had to suspend the church and conduct the delivery.’ (E3M2)*

This issue was also raised by community residents who perceived it to have deterred some women from seeking care.

*‘If they don’t have enough nurses in case someone goes into labor in the night so they have nurses that are at work in the night, also have nurses available in the morning and afternoons too, so that whenever you come you will be able to see the nurses. That’s what I have to say […] Like from today if government will agree to bring in more nurses to this place if the nurses here are not many and also bring other thing that will help them in carrying out their duties effectively. If it is done, then we would also be coming to the health facility.’* (E2FG2)

*‘Another issue is lack of enough staffs…for the run of 24 hours shift. […] they [midwives] are not enough to cater for the population.[…] We are seeking for additional staffs for the clinic as currently there are not enough health workers at the facility for a complete 24 hour shift […] the most important challenge is lack of adequate skilled health workers.’ (K1FG1)*

Another human resource constraint issue raised was the lack of doctors. In Enugu, midwives reported that their ability to deal with complications during pregnancy and labor was limited by the lack of a permanently stationed doctor at the clinic.

*‘We also need medical personnel, that is, a medical doctor on regular basis, though there are corps members that come occasionally.’* (E1M1)

This was also mentioned by community members who reported that the lack of consistent medical doctor coverage affected the confidence that they had in services at the clinic and their belief that delivery complications could be dealt with there.

*‘The most important thing for us is doctor; to ensure that doctors are always around; because if they are always around, the challenges around childbirth will be reduced. If in case of complications during childbirth…if it was only the nurses were around (our nurse are trying really, they are very hard working in terms of delivery), but if a doctor is around, if it were cases that the nurses cannot handle, the doctor will handle it himself because he is more experienced than the nurses.’* (E1FG1)

*‘You know that it is not all the illness that the midwives can handle. So the absence of doctor in this place is part of the drawbacks we are having here; because if there is doctor here, you will be confident that anytime you have illness, there is always a doctor to handle it.’* (E2FG2)

*Irregular payment of salaries and inadequate housing*

The most common challenge raised across the three states was the delayed (and in some cases complete non-) payment of monthly allowances. The extent of delayed payments had reached such a level that midwives in all three states cited it as a reason for wanting to leave the scheme. Midwives reported that they were four to six months behind in salaries and that the situation had gotten worse over time.

*‘When we started this midwifery service scheme, they were doing very fine. Federal government, […] they were paying us. Before the end of the months, they will pay us. But since last year, my brother is something else. They have owe us six months, seven months, five months, they will owe us, and when they will pay, they will just give us one month. Except last week that they just gave us four months’ salary, which they have been holding…’* (E2M1)

*‘We have problem with our… federal […] they don’t pay us, sometimes 4 months, 5 months, 6 months, before them pay us..’* (K2M1)

In addition to delayed salaries from the federal government, some midwives reported not receiving any allowances at all from the local governments.

*‘We are still protesting for the local government to be paying us. It is written in our deployment letter that they should be giving us supplementary allowances, and we have been going there for it. They keep asking us, ‘what is MSS?’ they said we are not their staff. The local government chairman even said that he is trying to pay those that he employed, let alone we. He said we are just here for supplementary, he was not the one that employed us.’* (E3M2)

*We are not being paid our remuneration. We were last paid [7 months ago]… we were supposed to be given stipend by the local and state government but we have never been given a kobo by the Kwara state government.[…] The local government was giving ten thousand naira before but after a year they stopped it so nothing, nothing. (Kw1M1)*

Another issue reported by midwives was with the provision of accommodation. They reported that housing was either not provided or that what was provided was inadequate.

*‘Do you know what? The house we are staying, there is no ceiling! At times we waste time at the hospital, so that it becomes cool, before we reach the abode. In the night, mosquitoes! Unless we enter inside mosquito nets. It is terrible!’* (K3M1)

*The accommodation is not ok. With the Ebola problem, we are living with bats. So we complained, and they are promising. And up till now, nothing has been done. (Kw3M1)*

This led to discontent and contributed to feelings of insecurity. In some cases it led to midwives living away from the health center, which affected their availability in the health facility at night.

*‘If there is an accommodation made available, we will stay and attend to patients. You don’t expect me to be sharing a room with somebody. I have passed that stage. You don’t expect me to go to toilet and keep squatting now, I have passed that stage. At times, you’d see snake coming out because the place is not fenced. People will come and leave, even at night you are not safe, you wouldn’t know what will happen at night because a lot of people come here. They invade this place at any minute of the day. But, if they had made things to look good, it is our responsibility to stay at work because we know that deliveries normally come in the night. When they come and they don’t see midwives, it doesn’t tell well of us.’* (E2M2)

This issue was also noted by community residents.

*‘In this our town, some of our midwives do not stay at the service of the women; especially when the women want to give birth because many of them do not live in this our …this thing…* [probably referring to having accommodation within the facility] *especially in emergency cases. A woman may be experiencing labor in the night, and she runs to this place, and sometimes. That’s why some when they come here and they don’t see some of them in the night, especially, the husband may rush her to any nearby…to some of these local …this thing…* [possibly referring to traditional birth attendants]*. I’m saying that if enough midwives will be provided here and they give them accommodation to see that some of them work…just like in the township, you know that some work in the night, and some work in the afternoon. Unlike in this place, they don’t do like that in this our area. When it is in the night, you will not get anybody. So in emergency cases, some of the pregnant women suffer.’* (E2FG2)

*‘Their houses really there are houses, not that there are none, but really they are in need of repairs. Because actually even ceiling there isn’t. Like during hot season, they really endure a lot of heat. There is no ceiling, no generator no fan that they can be using. Actually they face those problem, because they do complain.’* (K3FG2)

#### Poor clinic infrastructure and lack of supplies

The poor state of clinic infrastructure was noted as an important service delivery constraint. In Kwara state, for example, midwives reported that the poor condition of the facility hampered their ability to deliver care.

*‘That is supposed to be our office if you get there now half of it is just sand, even the doors to this clinic are not closing, they are eaten up by termite. The structure itself is bad.’ (Kw1M1)*

*‘It is not conducive for delivery because the roof of the delivery room has been blown off and there are not enough beds […] There is no room to keep patients for observation especially when it’s raining. We can’t admit for 24 hours. […] no toilets for patient use.’ (Kw2M1)*

The unreliable supply of electricity was cited by midwives in three health facilities, all in Enugu. They reported that this impacted their ability to deliver basic care during delivery and to use electrical equipment.

*‘You know, it is recently that they brought all the instruments needed for the delivery here. Could you believe that up till now that I am just seeing light for the first time? We conduct deliveries in the night with lantern. Where is it done in the modern world? How much does a generator cost? Yet, they find it difficult to provide a generator for this facility. How can you be delivering with lantern?’* (E2M2)

*‘We have Sonic K here, but we don’t use it because we cannot charge the battery.’* (E1M2)

Residents in these communities echoed the midwives concerns, noting that the lack of basic amenities such as electricity and water impacted on the midwives’ ability to deliver care.

*‘We urge the government to provide a standby generator for us at the health facility. Because sometime when they are working and there is light failure it equally affect their work.’ (Kw1FG1)*

*‘Then another one concerning water, because there is supposed regular supply of water especially at the delivery room; because if there is no water, there are certain things that the nurses usually do immediately after conducting delivery; they will use water to take care of the person who just delivered. So water supply is very important in our community – and good water supply. This will help in doing things when they are supposed to be done; because a woman may come in the night and after delivery and it may be that there is no water, and you know is not good that after delivery, the woman will remain unclean till morning.’* (E1FG1)

The lack of drugs, equipment, and other supplies were also cited as important challenges. Drugs were reported to be only periodically available in most clinics. This was perceived by one midwife to create a negative perception of the clinic in the minds of the women.

*‘Yes, at times we have some problem. For example, we don’t have the MAMA kits and there is no place to even buy a pad in this village. Because we told them to come as everything is free. So they come without anything. In cases where they need fluid and drugs we prescribed for them to buy at the pharmacy.’* (K1M1)

#### Poor integration with existing staff

For some older midwives who had previously been in retirement, having to work under less experienced and younger staff was a source of frustration. In one case, the converse was also true with one newly qualified midwife reporting that she found working under more senior midwives challenging because of their resistance to new ideas and practices.

*‘You know, we are the midwives and the people we work with are CHEWs [community health extension workers], and it is not so easy to work under your junior, because these CHEWs [community health extension workers] are also our junior […] Initially, I found it difficult because it is understandable when you are working with your fellow nurse, whether it is your junior or senior; but when you are working with somebody that is far lower than your own status, it is not easy, because you feel humiliated somehow.’* (E1M1)

*‘I think that one other problem again is the supply of drugs; drugs, enough drugs. The availability of drugs! It is not that for instance, if there is no doctor, and if there other people that may be standing in place of doctor, when you come here, the problem will now be that there is no drug.’* (E2FG2)

*‘And if they have anything to help to equip the health centers, please they should do. Like this our health center, we need foam, we need to change mattress in this health centers. But we have been complaining that to local government chairman, sorry HOD [head of department] and nothing has been done. So if federal government will help us, it’s good.’* (E2M1)

*‘I’m not happy about the equipment we use; we don’t have enough instruments. Have you complained to your higher authorities? Their response was that they will bring it.’* (K2M1)

*‘You know, for instance, if say that… you know for instance, you know we have… there are old staff, that feel they finished school in the [19]80’s 90’s, so when we are correcting them, for instance, we say, don’t use normal saline, don’t use this, don’t use that…they will say, [they] worked before us’* (K2M2)

## Discussion

The findings from the interviews and focus groups reveal important design, implementation and operational challenges that likely contributed to the program’s lack of impact. Some of the problems with the design of the program included the requirement that midwives be posted to different states. This mimicked the design of other government programs in Nigeria such as the National Youth Service Scheme, which is a compulsory year of community service for university graduates [19]. Though this redistributive feature of the program allowed implementers to address supply constraints in certain regions such as the north, which has lower levels of schooling and educational attainment [17], it contributed to midwives not wanting to remain long term in the Scheme, particularly for midwives posted to northern states. Another issue with the design of the Scheme was that it did not align well with the organization of the health sector in Nigeria. The MSS was managed by the National Primary Health care Development Agency (a Federal agency). They recruited and hired the midwives. However, primary health facilities are the responsibility of local governments whose funding comes from state budgets [4]. One of the consequences of this is that it was not always clear who was responsible for what (or whom). It also created a two-tiered system within primary health care clinics (and within states) with MSS midwives not having access to some of the same opportunities as state-employed health workers, contributing to dissatisfaction.

A key feature of the program was that it involved cooperation among the three tiers of government, federal, state, and local. Though the intention was to encourage a sense of joint ownership, in practice, state and local government officials often saw the MSS as a federal program that was foisted on the states without appropriate consultation and input, particularly during the planning and implementation stages, suggesting that federal implementers did not do enough to secure sufficient buy-in and cooperation from states and local governments. This was an important problem because the success of the program ultimately depended on contributions from each tier. This appears to have had negative cascading effects. Dissatisfaction led to disengagement, which likely contributed to many of the operational issues that we noted such as the irregular provision, and in some cases non-provision, of agreed midwife benefits by state and local governments. This, in turn, had negative effects on midwife satisfaction and willingness to renew their contracts and continue working for the scheme. These findings underscore the importance of engaging with critical stakeholders groups throughout all stages of an intervention, especially in the context of complex interventions such as the MSS, and having an iterative and ongoing process of engagement and feedback [12]. It is important though to note that the federal government also did not consistently fulfill its responsibilities, a problem which according to midwives had worsened over time. An example of this was the delayed payment of midwives’ allowances. At the time of data collection, midwives reported being owed several months worth of allowances.

MSS midwives also faced other important operational constraints such as deteriorating clinic infrastructure, human resource constraints, and lack of drugs and equipment. They reported that this affected their ability to delivery care. For example, unreliable supply of electricity hampered the use of equipment such as fetal monitors. These constraints also appear to have contributed to low uptake of maternal and neonatal child health (MNCH) services by households, both directly (because of provider availability for example) and indirectly by impacting perceptions about the quality of care available in the clinics. Though the MSS included a one-time provision of basic equipment and supplies, its focus was on alleviating human resource constraints (by deploying midwives). It therefore did not address some of the other concerns with clinic quality. Our data suggest that these factors contributed to deterring households from using clinic services. Other studies have shown that these factors play an important role in household demand for health services [7, 10]. Households also continued to face barriers to accessing care that were not addressed by the MSS, including challenges with getting to health clinics and referral facilities. We also find some evidence that factors such as lack of awareness about the importance of skilled care also continued to affect uptake of services.

The challenges noted in this study provide opportunities for program improvement. Near-term recommendations include better and more intensive engagement with state and local-level policymakers, timely and adequate compensation for midwives including provision of other job-related benefits such as proper housing and professional development opportunities, and local recruitment and posting of midwives (subject to availability constraints) to help with staff retention. We recognize though that given the realities of geographic distribution of health personnel, the posting of midwives to locations far from their states of origin/residence is likely to remain a feature of the program, at least in the short to medium-run. One solution that can be implemented in the near-term is to include wage adjustments for distant postings to compensate for greater relocation costs (under the current scheme all midwives are paid a uniform wage). This is similar in spirit to cost-of-living adjustments, ‘hardship’ allowances, or ‘danger’ pay, which recognize and compensate for differences between locations. In the longer-term, interventions to increase the supply of health personnel in shortage areas should be a focus of policymakers (for examples of such programs see [2]). Other long-term recommendations include interventions to address constraints faced by households such as costs associated with use of services including transportation costs, and investments in improving the quality of service delivery, for example improving clinic infrastructure and the availability of equipment.

This paper highlights the inherent challenge of effectively scaling up maternal and child health interventions [5, 6]. Studies of similar programs such as the Safe Delivery Incentive Program (SDIP) in Nepal [22] and the *Bolsa Familia* program in Brazil [15] have documented implementation challenges. For example a qualitative implementation study of the SDIP found long delays in disbursement of funds from the central government – in some cases by more than nine months – leading to beneficiaries not being paid on time, frustration at the district level at a lack of consultation by the central level during the planning process, and perceptions that the program did not make enough investments in improving the availability and quality of services [22].

We acknowledge that this study has some limitations. The qualitative interviews and focus groups (except for the federal policymakers interviews) were conducted in three purposively selected states. The experiences in these states may therefore not be reflective of experiences in other states. In addition the study participants are not a random draw from the population in these states. Appropriate caution should therefore be exercised in generalizing the study findings. We note, however, that we recruited a range of participants with different characteristics in order to increase generalizability. Another limitation of the study is that the midwives we interviewed were those who were currently in service; we did not interview individuals who had left the program, whose perspectives may be different. If anything, however, we would think that midwives who left were those who were more dissatisfied and would therefore likely have more negative perceptions of the program. In that sense our findings may understate the true state of events. Relatedly, the views of midwives in our study may be taken as reflective of the current state of events (and not of the overall program). However, it is important to note that we interviewed midwives with varying levels of program experience based on the length of time working in the MSS. On average, they had been employed for just over 3 years (the program had been in place for about five years at the time of data collection) suggesting that their views are likely to reflect the historical as well as the present context.

## Conclusion

As policymakers in low and middle-income countries continue to work towards improving maternal and newborn health, the lessons learned from programs such as the MSS offer important insights. Although we analyzed a specific program that naturally faced a number of context-specific challenges, our findings nevertheless yield insights that are of general relevance for health planners in developing countries. These include the critical importance of program design, particularly when programs are implemented at scale; the need to identify and involve key stakeholders during planning and implementation; the importance of clearly defining lines of authority and responsibility that align with existing structures where possible; and the necessity for multi-faceted interventions that address multiple barriers at the same time [24].

## Additional file

Additional file 1: File contains the semi-structured interview and focus group discussion guides used in the study. (PDF 134 kb)

## Abbreviations

ANC: Antenatal Care
CHEW: Community Health Extension Worker
LGA: Local Government Authority
MNCH: Maternal, neonatal and Child Health
MOU: Memorandum of Understanding
MSS: Midwives Service Scheme
NPHCDA: National Primary Health Care Development Agency
PHC: Primary Health Center
SDIP: Safe Delivery Incentive Program
UNICEF: United Nations Children’s Fund
WHO: World Health Organization
